# Early and effective use of ketamine for treatment of phantom limb pain

**DOI:** 10.4103/0019-5049.63632

**Published:** 2010

**Authors:** Harsha Shanthanna, Medha Huilgol, Vinay Kumar Manivackam

**Affiliations:** McMaster University, Health Sciences Centre 2U1, 1200 Main Street West, Hamilton, Ontario, Canada L8N 3Z5, Canada; 1 Department of Anaesthesia, Critical Care and Pain, Columbia Asia Referral Hospital, Yeshwantpur, Bangalore - 560 055, India

**Keywords:** Ketamine, NMDA antagonist, phantom limb pain

## Abstract

Treatment for phantom limb pain is difficult and challenging. There is often suboptimum treatment with fewer than 10% receiving lasting relief. Treatments based broadly on other neuropathic pains may not be appropriate for a clinical success. We report a case of phantom limb pain, which proved resistant to multiple analgesics, including opioids and continuous epidural blockade. Treatment with intravenous (IV) ketamine as an alternate day infusion, gave complete remission of phantom limb pain. This demonstrates an early and effective use of a potent NMDA antagonist for treatment of phantom limb pain. Mechanisms underlying phantom limb pain are briefly discussed.

## INTRODUCTION

Phantom limb pain is a unique neuropathic pain syndrome that develops following amputation in approximately 80% of the patients.[1] There are suggestions of different mechanisms accounting for phantom limb pain in its acute and chronic stages, leading to a possibility of different treatments being effective at different stages.[2] NMDA receptors are known to play a central role, in its development.[1,3] Ketamine, a potent NMDA antagonist has been successively used in many neuropathic pain conditions. However, there are no clear guidelines regarding its optimum use in phantom pain.

## CASE REPORT

A 12-year-old boy suffered severe polytrauma and sustained fractures, lacerated and avulsed wounds, primarily involving his limbs. His right leg, which had a threatening vascular injury, was debrided and kept under close observation. He underwent three procedures before a decision was taken to amputate his right lower limb, below knee. However an epidural was not performed, as sepsis was a consideration, and he was put on IV fentanyl infusion for postoperative pain relief.

**Figure 1:**
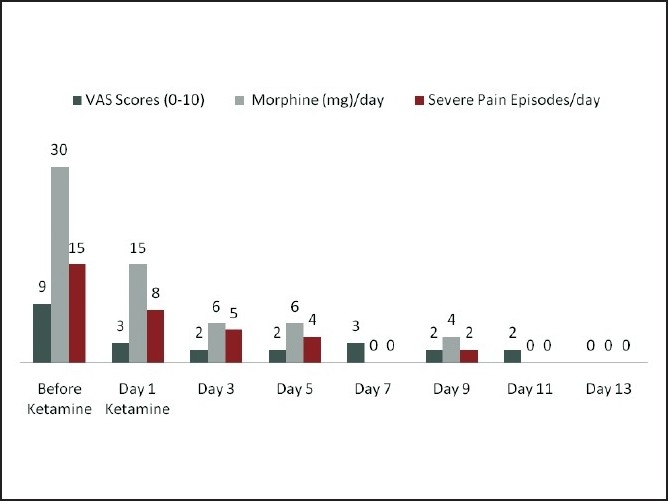
Record of pain score (VAS upto10), morphine use (in mg) and number of severe pain episodes (VAS >6/10); from the day before ketamine treatment to complete pain relief.

Subsequently he was put on oral analgesics and rescue doses of IV morphine. On the fifth postoperative day, he complained of severe pain from the non-existing part of his leg with pins-and-needle sensations from the stump. The pain management team was then involved in managing his emerging phantom limb pain. We found an extremely anxious boy with a history suggestive of phantom limb pain and sensations. The pain was not continuous, varied from moderate to severe in intensity (average Visual Analogue Score 8/10), with a shooting character, which went to burning at times. On an average there were 15 – 20 episodes of severe pain (VAS of more than 6/10) per day. Hyperalgesia and cold allodynia were also noted. The boy also had generalised pain due to other injuries. A lumbar epidural catheter was inserted aseptically and he was put on continuous epidural analgesia with 0.1% bupivacaine, along with oral ibuprofen, gabapentin 100 mg BD, and amytryptline 10 mg HS. This made a drastic difference, with his pain score coming down to 3/10 from 8/10. However, he complained of increasing pain on the third day of epidural infusion. After checking for a functioning epidural, gabapentin was increased to 150 mg BD. On the next day, as the pain became more resistant, he was put on rescue doses of IV morphine. On the fifth day, the IV morphine used was 30 mg, a decision was taken to discontinue the epidural. As the pain showed increasing resistance to routine analgesics and opioids, we decided to initiate IV ketamine. After an ineffective trial of 150 mcg/kg, we decided to go with 300 mcg/kg with 2 mg midazolam, in a 60 ml solution, given over three hours, in a monitored setting. The VAS score decreased to 5/10 after two hours and to 3/10 that night, without any side effects. We pursued the same dose with alternate day infusions for 12 days. The use of morphine decreased substantially as the VAS scores decreased consistently. The number of severe pain episodes also reduced [Figure 1]. After six infusions the boy had no recurrence of limb pain for nearly 48 hours, after which he was discharged home. Further follow-up up to six months showed no recurrence, without any analgesic support.

## DISCUSSION

This report demonstrates an early, appropriate, and effective use of ketamine for phantom limb pain, especially when it has been resistant to other forms of treatment. Phantom pain is known to occur in approximately 80% of amputees, in some part of their life time.[1] The onset and duration of pain varies widely. Although not proven, pre-amputation pain levels and anxiety probably play a significant role along with the quantity and quality of sensory input at the time of nerve injury.[1,4]

This boy had significant pre-amputation pain due to multiple injuries, giving rise to higher sympathetic discharge and a state of hyperexcitation to the nervous system. This had perhaps led to the early appearance of phantom limb pain and sensations. Various factors played a role in the pathogenesis of phantom pain. It was evident from various animal and human studies that complex changes occurred, which triggered neuropathic pain development; such as, hyperexcitability and sensitization of the NMDA receptors,[1‐3] wind-up phenomenon, neuroplasticity,[1] including alteration of neurosignature,[1,5] and reorganisation of somatosensory representation.[5] At the periphery, neuroma formation led to a continuous input of aberrant neural hyperactivity.[1] Animal studies have demonstrated the central role played by the NMDA receptor activation in nerve injury models and also modifications of those changes when it was pre-empted by NMDA antagonists.[3,6] With the view of reducing sensory input and decreasing C fibre sensitization, pre-emptive analgesia using regional blockade is advocated. A randomised controlled study found no difference in the incidence, when either epidural bupivacaine or morphine was used 18 hours before surgery or immediately after surgery.[7] Moreover, it was also seen that a local anaesthetic administered through an epidural did not completely abolish the peripheral sensory input and electrical activity below the threshold required to stimulate the spinal cord and brainstem, as shown by functional MRI.[6,8] Pre-emptive use of ketamine did not show much benefit in many studies[9] except with the study of Wilson *et al*.,[6] wherein, they used ketamine epidurally, and stated that the route of administration may be clinically significant in terms of efficacy. In our case ketamine was used as an early treatment rather than pre-emptively.

Aggressive pain management with ketamine, at a later stage, when the pain is quite established, has shown good results.[3,8] There were no side effects with the use of 300 mcg kg 1, given over three hours. Various doses have been used in other neuropathic conditions.[10] What determines its optimum use in terms of dose, frequency, and duration is yet to be established. An early and aggressive treatment, titrated to patient effect, may prevent supraspinal and cortical reorganisation changes.

In summary, phantom limb pain may not be always amenable to pre-emptive analgesia.

However, when symptoms do appear, it may be appropriate to introduce early therapy with proven NMDA antagonists, so that sensitization and long-term changes are prevented.
